# Islam and cannabis: Legalisation and religious debate in Iran

**DOI:** 10.1016/j.drugpo.2018.03.009

**Published:** 2018-06

**Authors:** Maziyar Ghiabi, Masoomeh Maarefvand, Hamed Bahari, Zohreh Alavi

**Affiliations:** a Ecole des Hautes Etudes en Sciences Sociales, 54 Boulevard Raspail, 75006, Paris, France; b Substance Abuse and Dependence Research Center, University of Social Welfare and Rehabilitation Sciences, Tehran, Iran; c Department of Social Work, University of Social Welfare and Rehabilitation Sciences, Tehran, Iran

**Keywords:** Religion, Cannabis, Legalisation, Regulation, Iran, Middle east, Islamic law, History of cannabis

## Abstract

Iran is currently discussing cannabis and opium regulations, which could bring a legalisation of drug consumption through a state supervised system. The article engages with the question of cannabis by looking at the legal interpretation of religious authorities in the Islamic Republic of Iran. The choice of Iran is justified for several reasons: firstly, Iran has a long history of drug use and cannabis has been part of the country’s intoxicant traditions since times immemorial; secondly, the Iranian state is unique in that it combines religious exegesis with political machination through official channels; finally, among all Middle East and Islamic countries, Iran is at the avant-garde in experimenting in the field of drugs policy which makes an excellent case for the study of cannabis regulation. The article is the result of a direct engagement with Iran’s leading Shi’a authorities, the *maraje’-e taqlid*, ‘source of emulation’. The authors redacted a list of eight questions (*estefta’at*) about the status of cannabis in Iranian society. It questioned cannabis’ legality in Islam, its potential medical use, the feasibility of domestic production and other relevant aspects of its social-religious life. Based on the responses, the authors analysed the difference in opinions among the religious scholars and speculate on the possibility of policy reform. Given the dearth of scholarly work about illicit drugs in the Islamic world, about which many readers might not be familiar, the article opens with an overview of the place of cannabis in the history of Islamic societies. It discusses terminological ambiguities, references in religious texts and traditions, and the general interpretations within Muslim religious schools of thought. Then, it discusses the status of cannabis in contemporary Iran before tackling the responses provided by the religious scholars. Eventually, the paper puts forward reflections about the potential implications for future policy developments on cannabis.

## Introduction

Middle Eastern states and the Islamic world are known for adopting strict codes of prohibitions. These apply to sexual norms, such as premarital sex, social practices such as gambling and consumption behaviour as in the case of alcohol and narcotic drugs. Narcotic drugs however are hardly exceptional to Islamic societies and/or the Middle East. Across the globe, prohibition of narcotic substances has been a common trait of the 19th and 20th centuries (Courtwright, 2009; Campos, 2012; Carrier & Klantschnig, 2012; Mills, 2003; Robins, 2013; Safian, 2013). Countries as different as the United States, Britain, China, the Soviet Union (and later the Russian Federation) as well as almost every Third World, Global South state have adopted forms of regulation and prohibition of narcotics (Chouvy & Meissonnier, 2004; Chouvy, 2009; Windle, 2016). Yet, change is under way in this sphere. Since the 2000s, several countries in the Western hemisphere have taken steps towards changing the regime of prohibition of formerly illicit drugs. In particular, cannabis has been the object of these undertakings. Portugal, Uruguay and a number of US states have adopted policies that regulate the use of cannabis among the population (Domoslawski & Siemaszko, 2011; Pardo, 2014). Other states are in the process of evaluating and updating their model of cannabis regulation, such as Spain, Italy and, surprisingly, the Islamic Republic of Iran.

The debate around cannabis’ status today is regarded as a turf of Western policy and scientific circles. The Global South and, particularly, the Middle East, where once the drug had its roots and tales of inebriation, is regarded as static. The Middle East is seen as a status quo region with regard to drug policy reform (Robins, 2013). Yet, Islamic societies have had long and animated histories of debate around the merits and evils of cannabis. Persian and Arab scientists, religious scholars, poets and historians have evaluated the place of cannabis in their respective social milieu (Afsahi & Darwich, 2016; Nahas, 1982). They preceded by many centuries the drugs policy circles that are active in the 2000s. From this perspective, the potential of debate and change around the status of cannabis in the Middle East and Islamic World is high, even when compared with the social conservatorism of many Arab countries.

Hence, the article engages with the question of cannabis by looking at the legal interpretation of religious authorities in the Islamic Republic of Iran. The choice of Iran is justified for several reasons: firstly, Iran has a long history of drug use and cannabis has been part of the country’s intoxicant mores since times immemorial; secondly, the Iranian state is unique in that it combines religious exegesis with political machination through official channels; finally, among all Middle East and Islamic countries, Iran is at the avant-garde in experimenting in the field of drugs policy (Ghiabi, 2017; Razzaghi, Nassirimanesh, Afshar, & Ohiri, 2006), which makes an excellent case for the study of cannabis’ legal status and prospects of reform.

The article is the result of a direct engagement with Iran’s leading Shi’a authorities, the *maraje’-e taqlid*, ‘source of emulation’, who are religious scholars legitimated with the interpretation of religious rules. The authors redacted a list of eight questions (*estefta’at*) about the status of cannabis. The questionnaire touches upon cannabis’ legality in Islam, its potential medical use, the feasibility of domestic production and other relevant aspects of its social-religious life. Based on the responses, the authors analyse the difference in opinions among the religious scholars and speculate on the possibility of policy reform. Given the dearth of scholarly work about illicit drugs in the Islamic world, about which many readers might not be familiar, the article opens with an overview of the place of cannabis in the history of the Middle East with an especial focus on Iran. It discusses terminological ambiguities, references in religious texts and traditions, and the general interpretations within Muslim religious schools of thought. Then, it discusses the status of cannabis in contemporary Iran before tackling the responses provided by the religious scholars. Eventually, the article puts forward reflections about the potential implications for future policy developments on cannabis.

## Method

The article is based on a combination of archival research, literature review and questionnaires and interviews on the subject of cannabis’ legal and religious standing in Iran. For the historical part, we made use of the National Archive in Tehran and on collections of published material in Persian. We also carried out an overview of the published material in English and French and integrated that with our own archival collection. Following that, we surveyed the legal regulations with regard to cannabis in modern Iran and paid attention to the developments under the foundation of the Islamic Republic in 1979. This included an engagement with the policy debates over the last five years, which touched upon possible cannabis law reform. Finally come the most original side of the project which is the engagement with clerical authorities:

The position of clerical authorities today is unclear with regard to cannabis and potential medical use of it. Convinced that their position could have an impact for current policy debates, we approached nineteen leading *marja‘s* based in Iran (and Iraq) through a set of eight questions on cannabis. The selection was based on the prominence of the religious figures and included *marja‘s* in Iraq since the holy cities of Najaf and Karbala in Iraq are home to prominent figures which hold influence also in Iran.^1^ Prominence here is defined on the basis of public acknowledgement of the *marja‘s* standing, a fact that although it may sound disputable has been upheld rather uncontested over several centuries.^2^ Before engaging the *marja‘s* the team prepared a text on current scientific knowledge about cannabis. This synthetic text in Persian illustrated the major lines of the debate about harms and uses of cannabis as reported in the current scholarship. The text was annexed to the questionnaire sent to the clerical authorities who could use it in case they required further details about the elements included in the questions. Table 1 illustrates the questions asked to the *marja‘s* indicates the names and profiles of the scholars involved in the study, together with their responses to the questions. Not all the *marja‘s* responded to the questionnaire and we avoid speculation about this. One should add that these scholars deal with hundreds if not thousands of questions every day; the very large mole of questions that reach the *marja‘s* may impact on their response capacity. Moreover, those *marja‘s* that hold important political roles in the Iranian state may have avoided responses to the questions in order not to weight on legislative process. This may especially hold water in the case of the Supreme Leader, Ayatollah Ali Khamenei.

**Table 1:** Questions sent to the Marja's.

Questions
1. Is the consumption of cannabis and its derivatives permitted?
2. If cannabis and its derivatives are consumed for treatment of diseases, what is the judgement?
3. Is the cultivation of cannabis for the purpose of production of legal medical and pharmacological products permitted?
4. Is cultivation and production of cannabis for export permitted?
5. Some people believe that hashish is *najes* (impure). Is hashish impure? If yes, why?
6. If cannabis and its derivatives (e.g. hashish, marijuana, etc.) are used for medical consumption, what should the Muslim do in the month of Ramadan? Can he use it while he is fasting?
7. If the person has used cannabis and its derivatives (hashish, marijuana, etc.) is his prayer valid?
8. Are artistic products, such as films, songs, etc., which show cannabis use, permitted?

**Fig. 1:**
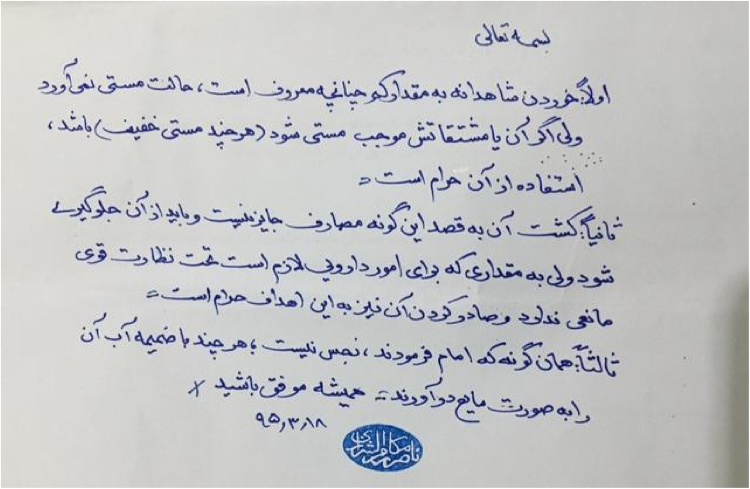
Ayatolllah Makarem Shirazi's response.

The engagement with the *marja‘s* occurred through the mailing (and e-mailing) of questions (*estefta‘at*) to the *marja*”s central office. The office took the questions in charge and provided a response, undersigned by the *marja‘* (Fig. 1). The format of response is synthetic and the *marja“s* don”t provide specific reasons for their opinions. This style is based on the acceptance of their authority legitimatised on an established career as an Islamic jurist. In the article’s last section, the reader can find a summary of the questions and the responses and an analysis of these.

## Historical background

*Hum*, *homa*, *soum*, *sama*, *giyah-e javidan* (‘the herb of the immortal’), *giyah-e moqaddas* (‘sacred herb’), *khadar* (‘green’), *kimiyah* (‘alchemy’), *qanaf* (‘hemp’), *zomord-e giyah-e* (‘smerald herb) are among the many expressions given to cannabis in the Iranian plateau (Gnoli & Saʿīdī Sīrjānī, 1988; Rabeie, 1998). More popular names are *hashish*, the Arabic word for “grass”, *bang*, an Avestan term indicating whole cannabis residue, or the Persian word for ‘grass’, ‘*alaf*. In contemporary times, other words have added up to these: *marijuana*, *sabz*, *jay*, *charas* and *gol.* But the name that has entered the Persian language is *shahdaneh*, “the royal seed”. The word is currently used to refer to cannabis both as a plant, a seed and a drug.

Prior to the Islamic era, the plant was cultivated and used in rituals by Zoroastrian priests. In one of the Avestan hymns contained in their sacred book, *Gat-ha*, reference to the defence of the environment and care of the ‘sacred plant’ is thought of ceiling a reference to cannabis (Rabeie, 1998, p. 19). Reference to cannabis for rituals and spiritual performance is abundant also during the Islamic era (651–…). Hashish, in particular, was known to be a favourite substance starting from the 13th century Mongol conquests. The Sufi sect of the Qalandars which originated in Khorasan (Eastern Iran) made of hashish consumption (as well as wine drinking) a hallmark of their public behaviour (Matthee, 2005, pp. 40–41). The *hashish* pipe, up to today, is referred to as *nafir-e vahdat*, ‘the trumpet of unity [with god]’ (Neligan, 1929). Dervishes, the mendicant Sufis, have also been known for their use of bang and hashish, for instance in the form of a yogurt and cannabis preparation called *dugh-e vahdat*, ‘drink of unity’.

These are few references to the presence of cannabis in the history of the Iranian plateau. Less plausible are the accounts borne out of Marco Polo’s voyage which popularised the figure of Hasan-e Sabah and his volunteer army of *hashishiyun*, ‘those on *hashish*’, in Alamut (from where many believed the word ‘assassin’ derived) (CASTO, 1970). This tale became an Orientalist trope that mediated the diffusion of cannabis in the Mediterranean and late across the West, with resonance in the travel accounts of European orientalists. However, cannabis was not simply a drug of religious deviance and heterodox spirituality. It had an established place within the pharmacopeia of Iranian civilisation. In *The Canon* of the Iranian scientist Avicenna recommended it as a useful analgesic for headaches (Gorji & Ghadiri, 2002). If opium (*taryak*) did not produce relief, the Iranian doctor suggested to take ‘ambergris, aloe wood, juniper and poppy head, saffron…of each one quarter gram complete; and of the chief ingredient of that which dreamers call “the mysteries” [*hashish*] a weigh equal to all…mixed into a mass with honey. Take of it occasionally as Sufis do’ (Neligan, 1929). The physician al-Razi (Rhazes) indicated hemp leaves as cure for ear problems, dandruff, flatulence as well as epilepsy (Nahas, 1982).

Therefore, in the medical practice of Muslim societies, especially in Iran, cannabis has historically been used as analgesic, appetite inductor, euphoric and sexual inhibitor (although prolonged use was known to diminish sexual impulse) (Peters & Nahas, 1999). This secular knowledge about the use and abuses of cannabis was equally enriched by the debate about its legal status among Muslim religious scholars. This medicalised debate on cannabis certainly benefited from the lack of explicit prohibition in the Islamic primary sources. While the prohibition of wine is an agreed matter based on the explicit Koranic forbiddance, references to hashish, cannabis and other hemp derivatives are absent from the sacred text. This void opens up the possibility of interpretation among legal scholars with results that are not always unanimous, as this article discusses.

Scholars have struggled to find early accounts on cannabis and hashish at the time of the Prophet Muhammad (571–632). There are several accounts (*hadith*) among theologians, but none of them is considered accountable or having a strong transmission line (*silsilah*). One reports the Prophet saying, ‘There will come a day when people will consume a substance called *banj* [*bang*]. I avoid them and they avoid me’ (Tabarsi, 1988). Another tells: ‘Salute on the Jews and the Nasiri [Christians], but do not salute on the bang smokers’ (al-Hindi, 1979; Tabarsi, 1988). Both accounts seem to surface at a time when bang and hashish have gained popularity in the Middle East, especially in Iran. They may be expression of heightened tensions towards public intoxication of cannabis around both 16th century and 19th century when these substances were unsystematically praised and punished (Matthee, 2005). Contemporary scholars rarely base their judgement on these sources; instead, they privilege hermeneutical approaches that can be indirectly related to cannabis consumption.

The lack of Koranic reference stimulated the mind of religious scholars in interpreting the status of cannabis. Given the void in the hermeneutical sources, scholars judged the validity (*halal*) or prohibition (*haram*) of cannabis use based analogy (*qiyas*). Wine (*al-khamr*) is the comparative element taken into account, but most scholars disagree on equating wine with cannabis. A widely accepted account (*hadith-e hil*) says, ‘Everything is allowed for you [*halal lak*] until you learn it is forbidden…’. Hence, cannabis does not carry a total prohibition among most Muslim scholars (Safian, 2013). Allameh Helli (1250–1325), a leading scholar, said, ‘for the poison that derives from the herbs [*hashish-ha*] and the plants, if it has benefits [*manfe’at*], its sale and trade is not an issue. If it does not have benefits, then it is not permitted’ (Rezapourshokuhi, 2002). In another source, the scholar asked from one of his students whether hashish is intoxicant and harmful and if it is forbidden, Helli responds, ‘What is known among the people is that hashish is intoxicant, so eating it is prohibited not because it is harmful to the body but because intoxicates...Despite the prohibition of hashish, it is not impure [*najes*] because impurity is specific to alcoholic spirits [*musakkerat*]’ (Rezapourshokuhi, 2002). Shahid al-Awwal, another prominent Shia scholar from Damascus, states that almost all scholars who have preceded his era or his contemporaries agreed ‘that plants known as hashish have been judged as prohibited’ (Rezapourshokuhi, 2002). Nonetheless social and medical remained unhindered by legal constriction, except for sporadic instances due to the rulers’ changing ideas about hashish (Matthee, 2005).

One interpretative category that may be particularly relevant in relation to cannabis use for medical reasons is that of ‘emergency’ (*zarurat*). Scholars may allow believers to use or to perform generally prohibited substances or acts if these are deemed necessary in situations of emergency, or absolute necessity (Schirazi, 1998). For instance, if a believer founds himself with great thirst in the desert and the only available drink is wine − a forbidden drink for Muslims – then he/she is allowed (indeed he/she is obliged) to drink wine in order to save his/her life. So, if cannabis is useful for the health of a person, especially under serious risk, it can be used even when considered prohibited. This approach is legitimated based on a Koranic verse (*al-kul maytah*) and an accepted tradition (*hadith-e raf‘*) which reiterate that forbidden acts are allowed in times of emergency, if they can be useful and save lives (Ghiabi, in press). The primacy of life over religious prohibition is thus generally sanctioned. In practice, however, the use of “emergency” in interpreting the law facilitates the approval of otherwise unacceptable behaviours. In light of these elements, the debate around cannabis in contemporary times has gone through a great dynamism, including with proposals of reform of the current prohibitionist regime.

## From cannabis prohibition to cannabis regulation in Iran

It is with the establishment of the modern state (1920s) and, especially, with the foundation of the Islamic Republic in 1979 that prohibition of drugs, including cannabis, become more stringent. In the first part of the 20th century, cannabis was a primary concern to Iranian policymakers (and religious scholars). Public anxiety has generally been manifested towards other mind-altering substances, such as alcoholic drinks and opium. The latter, in particular, was the object of a first state-led monopoly during the 1920s and a total prohibition later in 1955 (Ghiabi, 2018a). Popularly accepted within Iranians’ mores and an integrated part of the traditional pharmacopeia, opium was re-interpreted during the modernisation process as a backward habit and a highly addictive substance. This prohibitionist impetus coincided with the international effort, sponsored by the United States, to control (and later eradicate) opium culture (Musto, 1999). This new vision was eagerly espoused by Iranian lawmakers with the hope of demonstrating their conformism with Western modernity (Ghiabi, 2017). Cannabis was not included in Iran’s first drug laws and its control remained mostly a question of moral condemnation led by public intellectuals and religious zealots. Among social groups seen as principle consumers were university graduates returning from studies abroad and the dervishes who begged and smoked hashish with little care about mainstream morality (Gnoli & Saʿīdī Sīrjānī, 1988). In fact, the first Penal Law of the Pahlavi era, approved on January 27, 1926, referred exclusively to the crime ‘of opium, *charas* and *bang* public intoxication [*motejaher*]’ (Penal Law, 1926). The punishment were severe when one considers that previously there stood no fine: from eight days to three months incarceration or the payment of two to fifty *tuman*.

Despite pressures from the international drug control machinery, which had adopted measures to supervise and control cannabis, the Iranian government ignored the matter (Bouquet, 1950). Only on June 22, 1959, Iran’s Senate approved the first law that made cannabis illegal. Without any specific discussion of the matter, the law established that the provisions that apply to opium, following the 1955 opium ban, should now apply to cannabis too. This ban laid the ground for Iran’s signature of the 1961 Single Convention on Narcotics Drugs.

It was with the establishment of the Islamic Republic in 1979 that the Iranian state adopted a staunch and uncompromising approach vis à vis all mind-altering drugs, including cannabis. This move made the Islamic Republic to adopt among the most powerful measures to control and repress drug trafficking and use, under the banner of Islam rather than that of the international drug control regime (Ghiabi, 2015). The first drug laws were approved by the Council for the Expediency of State (*Majma‘-e Tashkhis-e Maslahat-e Nezam*) in 1988 at a time of great political quakes. With regard to cannabis, the law established that “the cultivation of cannabis [*shahdaneh*] for the purpose of narcotic production” is illegal. The penalties for cannabis were equal to those envisioned for opium production and they contemplated, in extremis, the death penalty (Anti-Narcotic Law, 1988).

While the Iranian authorities successfully eradicated poppy cultivation during the 1980s, the cannabis plant was harder to get rid of. Spontaneous flowering of cannabis is not a rare sight across the Iranian plateau and given that the government’s priority remained to curtail the flow of opiates − in consideration of the heavy toll of opium and heroin use – cannabis use remained partly an issue on the background. By the 2000s, in line with global consumption modes, larger numbers of Iranians had acquired a renewed taste for cannabis and its derivatives. The traditional hashish smokers, popularly known as the *bangis*, have in part been replaced by urban marijuana smokers, who belonged to the modern wealthier end of the social spectrum. This shift has signified also that cannabis is now obtained not only through illegal drug networks which travelled westward from Afghanistan, the country disposing of the highest quality of hashish, but also domestically. At times, cultivation of cannabis would occur in the balcony and terraces of urban centres, the Iranian climate being ideal for such agricultural endeavours (*Fararu*, July 27, 2014).

Cannabis use surfaced in the public eye when the head of the Drug Control Headquarters, Iran’s umbrella organisation for illicit drugs, affirmed that a new drug, *gol* (‘flower’) had become the second most common consumed drugs after opium (*ADNA*, July 3, 2017). *Gol* indicates the flowers of cannabis plant, which contain a higher level of THC and therefore produce stronger psychoactive effects. This was similar to the trend in Western capitals where cannabis potency has progressively gone up in part due to the governmental clampdown and in part in response to users’ preference (EMCDDA, 2017). In response to this development, Iranian drugs policymakers have been discussing possible reforms that would update the old-fashioned prohibitionist regime. One important case is given by the proposal to regulate through state monopolies the cultivation of the poppy and cannabis plant. This step would enable the supervision of legitimate opium and hashish consumption, which would occur according to specific limits about age, medical status and spatial location. Although the proposal has not yet been approved, it has been discussed in the highly influential Expediency Council (*ADNA,* September 12, 2015). It has been taken into account and promoted beyond governmental venues. During the International Conference on Addiction Science that took place in Tehran in September 2015, leading scholars in drugs policy and public officials organised a stand-alone panel that focused on regulation and legalisation of cannabis, putting forth models to be implemented in Iran.

## Analysis: religious interpretation of cannabis

Legislative reforms and policy developments are influenced by multiple institutional and knowledge agents. Drugs policy, too, operates through various lines of enquiries. Religion, understood as a body of traditions, ethics and dispositions, is one important aspect. This of course is relevant in the context of the Islamic Republic, where religion stands at the foundation of the political order.^3^ There are several reasons why the role of religion is key to the study of public policy in Iran. Firstly, the Islamic Republic is a political machine that combines secular institutions of government – such as anti-narcotics agencies – with religious forms of political formulation. The latter is best exemplified in the fact that Islam and Islamic law, as developed through the Shia Ja’fari School, are the base of the Constitution as written in 1980 (Schirazi, 1998). This means that laws and policies adopted by the state have to go through a process of evaluation that ensures their religious validity. When parliament approves a law, the text goes through an evaluation from an ad hoc body named the Guardian Council, which acts as a Constitutional Court. The Guardian Council assesses the laws based on the accordance with Islamic jurisprudence (*fiqh*). If the law is considered against Islamic law, it is rejected and sent back to parliament.^4^

Secondly, Shia Muslim refer to a *marja*‘, a “source of emulation” who is knowledgeable about religious affairs, to regulate the everyday religious practice. This person is a learned scholar, specialised in religious exegesis, with an undisputed authority among its peers. There are generally many *marja*‘*s* at the same time, so Muslims are free to choose whom to follow. The interpretation of the *marja*“ is the ‘final word” on an issue (Walbridge, 2001). In modern Iranian history, there are examples attesting to the social power of these religious figures. In 1890, the Iranian government under the Naser al-Din Shah signed a concession that granted a Tobacco monopoly to the British Empire. Mass protests erupted in the cities and a leading Shia authority, indeed a *marja“*, emanated a *fatwa* (a religious opinion) calling for the boycott of all tobacco products (Keddie, 1966). The boycott was so successful that it is said that of one the Shah”s wives told the shah ‘that she and those around her would avoid the use of tobacco because the *marja“* had declared its use religiously forbidden”. As Walbridge suggest, the shah’s wife listened to the *marja‘* rather than the husband (Walbridge, 2001).

Following the establishment of the Islamic Republic, the role of the *marja*“ has gone through a double process. On the one hand, it has gained prominence, increased its financial tutelage, and found itself closer to the political machine (whereas historically religious authorities had been quietist vis à vis power). On the other hand, they have seen their leverage eroded by the authority of Iran”s head of state, himself a religious scholar (*faqih*). This coexistence allows Iranian citizens (of Shia faith) to seek the opinion of their *marja‘* on matters of their concern, while being still expected to respect the laws of the state.

Religious interpretation has been the engine for radical change in the last three decades. For instance, Ayatollah Khomeini, the founder of the Islamic Republic, declared the legality and acceptability of sex change surgery. His religious response enabled people seeking gender reassignment, to find the support of the Iranian state in the process. All costs and assistances are today covered by state institutions and transgender people are fully recognised in their rights (Najmabadi, 2013). Another example is provided by the current Supreme Leader, Ali Khamenei, who has published his official opinion with regard to advanced fertility treatment. He believes that treatments such as in-vitro fertilization, including practices using donor eggs and donor sperm, are allowed and in line with Islamic jurisprudence, a position that puts him far ahead of many regulations worldwide (Clarke, 2007).

In the field of drugs policy, the *marja“s* have expressed condemnation of drug trafficking and called for state support to solve ‘the question of drug addiction” (DCHQ, undated). In the 2000s, harm reduction measures were approved and adopted nationwide, with the ideological support of leading clerics. The religious authorities approved of practices such as needle-exchange and distribution, methadone maintenance and other generally controversial measures. Recently the Iranian government has approved the opening of two safe injection rooms in Tehran (Ghiabi, 2017). No clerical opposition was manifested towards this project.

The responses that the *marja‘s* provided to the questions (Table 1) are reported in the Appendix A.

## Based on the Appendix A, one can come up with a number of analytical considerations

First, for the majority of the religious scholars questioned, cannabis is not *haram*, that is to say that it is not totally forbidden. This is no minor result if one takes into account the uncompromising ban on drug use currently in place in Iran. The fact that, from an Islamic law perspective, the substance has an ambiguous status means that discussions around its potential regulated administration can gain legitimacy in the public debate. Regulation of administration is what the large majority of the *marja‘s* suggest with regard to this substance.

Secondly, and crucially, the majority of the scholars is of the opinion that if cannabis is used for medical purposes, which must be demonstrated and justified through scientific and medical research, there is no ban on its use, as long as it is justified for reasons other than intoxication and inebriation. Some of the scholars argue that, given the health risk that the substance might cause, its administration must occur with a strong supervision of the state and with caution. In a nutshell, one can infer the structure of a state regulated model for medical cannabis. The *marja‘s* do not specify what does state supervision signify, as organisational aspects are the turf of political administrators and not of the clerical jurisprudence. One could speculate that by state supervision it is intended a strict control on production and sale of cannabis, according to specific rules regulating consumption and administration.

Thirdly, cultivation of cannabis for pharmaceutical production is regarded as legitimate for the majority of the scholars. Should the Iranian government take steps towards the cultivation of medical cannabis, as it is done in many countries, religious authorities would not pose objections as long as this is done with careful supervision. Some, nonetheless, would object on the ground that the substance is prohibited in itself. In principle, production and cultivation is not allowed if the objective is to transform the substance into something regarded as forbidden. That implies that, in hypothesis, cannabis could not be exported to countries where it is illegal. The legal argument adopted by the *marja‘s* insists on the respect of the laws in place in the country.^5^

Fourthly, one wonders, for instance, whether cannabis with a very low THC levels (and high CBD) would qualify as a non-intoxicant substance according to what reported in the Appendix A. If that is the case, then its consumption could be regulated with no religious concern because the substance does not intoxicate the consumer.

Finally, none of the scholars considers cannabis a fully legal and permitted substance according to Islamic law. Its legality, six out of eight argue, is subject to conditions, circumstances and limits (Table 2).

**Table 2:** Marja's and Cannabis Interpretation.

Cannabis is not allowed	Cannabis is allowed for medical reasons and under state supervision)	Cannabis is allowed
Hossein Mazaheri Esfehani	Naser Makarem Shirazi	
Mohammad-Reza Modaressi Yazdi	Asadollah Bayat Zanjani	
	Musà Shobeyri Zanjani	
	Abul-Karim Musavi Ardebili	
	Mohammad ‘Alavi Gorgani	
	Ali Sistani	

The second part of the questions (5–8) referred to how cannabis use affects religious practice for Muslims. The question on impurity (*najes*) is significant because it bears upon cannabis users and their relation to the rest of society. Impure substances and people who get in contact with them are to be avoided. It is said that *everything is pure for Muslims, unless proven otherwise*. All the scholars agree that cannabis and its derivatives are pure (that is to say, are not *najes*), because they do not belong to the list of impure substance that has been redacted over the last ten centuries.

As for medical use of cannabis during the month of Ramadan, most of the scholars suggest that it is forbidden and it breaks the fasting and should therefore be avoided. However, one of them, Ayatollah Ali Sistani, one of the most influential *marja‘s* worldwide, declares that it is allowed for the sick to use medical cannabis if not using it represents a harm on the person’s health. For most of the scholars, praying is valid while using cannabis, as long as the person praying understands what he/she is saying.

While overall the *marja‘s* maintain a conservative standing on the question of drug use, when asked to respond on the religious validity of cannabis use, for instance, for medical reasons, they demonstrate a level of pragmatism, that arguably could bestow some legitimacy on proposal of cannabis law reform.

## Conclusions

Cannabis has been long debated in the Islamic world. Despite most Muslim majority countries today enforce a strict prohibition on its use, there are countries such as the Islamic Republic where reform of cannabis laws are under consideration. The drug policy community has ignored this development and, in this regard, has reinforced Orientalist misconceptions about the inability of non-western countries to achieve meaningful change (Robins, 2013). Proof of this Orientalist gaze towards the situation of drugs in the Middle East is given by the absence of meaningul studies of the region’s drugs policy or, for that matter, drugs history. More often, prohibition of narcotic or stimulant substances is justified as an axiom of Islamic prohibitions on intoxication (Kamarulzaman & Saifuddeen, 2010; Robins, 2013). Yet, as this article shows, Islamic jurisprudence and, especially, shi‘ate jurisprudence has adopted a flexible and open-ended approach, for instance, with regards to cannabis. More interestingly, the case of Iranian drug law reform suggests that the process has engendered from a domestic debate around drug policy and not from the global reformist movement active in North America. Of course, ideas and models are evaluated by indigenous stakeholders on the global platform, but much of the witty-gritty transformations take form in continuity with historical debates around drugs policy.

As the first part of this article described, debates about cannabis within the Middle East and Iran have been vibrant for several centuries now. The experience of medical cannabis as well as the traditions of counterintuitive religious interpretation with regard to social and medical questions (e.g. status of transgenderism) set the background of today’s debate. In the second part of the article, instead, we tackled how the political order of the Islamic Republic, which has generally been described as conservative and isolated from international debates (also through sanctions), has been capable of promoting stimulating debates around cannabis regulation. The article focused on a peculiarity of the Iranian system: the coexistence of political and religious institutions. In the field of drugs policy, the article reported how religious scholars are not inherently against the regulation of cannabis. The majority of them accept forms of regulation and argue that cannabis is not totally forbidden in Islamic law.

Beside the value of this statement per se, the interpretations of the *marja‘s* is significant in light of the ongoing debate about cannabis legalisation in Iran.^6^ Already in 2016, a reform of the national drug laws indicated that, in the coming three years, the Iranian government should take control of the drug market with the objective to cut off the connection between drug trafficking networks and consumers (ADNA 12 September 2017). If taken seriously, this proposal would establish legal cannabis cultivation and distribution under some form of state supervision, which would need to be discussed at parliamentary and ministerial levels. It will not lead to a legal market of cannabis and neither the creation of Dutch style coffee shop system. In light of the religious input on this debate, which however we should not overemphasize, the potential model could be in the guise of a regulated distribution, possibly medicalised, and initially only for domestic use.

To the surprise of international observers and drugs policy scholars, Iran might be the next country to regulate cannabis.

## Conflict of interests

None.

## Figures and Tables

**Table:** 

Ayatollah *(Marja‘-e Taqlid*)	Responses to Questions
Naser Makarem Shirazi	(he responded discursively):
	‘Firstly, eating cannabis, for as much as we know, in small quantities does not produce intoxication, but if cannabis or its derivatives produced intoxication (even small intoxication), its use is haram.
	Secondly, its cultivation for consumption is not allowed and must be countered. But in quantities necessary for pharmaceutical reasons under strong supervision, it is not prohibited. Its export too for this purposes is forbidden.
	Third, as Khomeini said, hashish is not impure.’
Asadollah Bayat Zanjani	1. If it doesn’t have significant effects or side-effects, there is no problem 2. On the question’s hypothesis, there is no problem. 3. If it is programmed and can be controlled and is not abused there is no prohibition. 4. If it is for valid and reasonable objectives, it is not a problem. 5. It’s not impure unless dissolved in a drink. 6. If it is eaten or drank, it has the same regulation as the other edible things. 7. If during the prayer you are intoxicated (*khomar*) and do not understand what are you saying, then the prayer is not valid. 8. If it is exclusively for advertising cannabis use, then it is not allowed.
Musà Shobeyri Zanjani	1, 2, 4. If it has a fundamental harm for the body or if it causes corruption to society, it is not allowed. Otherwise, if there is a law on tis issue,…, it must be respected. Its export too if it is intended for illegal consumption is not allowed.
	3. Based on the question’s hypothesis, it is not forbidden.
	5. It’s pure.
	6. If it is a booster/regenerator, then it is not allowed. If it is a powerful pleasure inducing one, based on caution, it is not allowed.
	7. It’s valid.
	8. It depends whether what they advertise is legal or illegal use.
Abul-Karim Musavi Ardebili	1. It varies depending on type of the drug, way of consumption and use. If it is useful for the human, and it is not narcotic, there is no issue. 2. If it is not illegal use then it is not an issue. 3. Cultivation of cannabis for legal use poses no issue, but if there is potential illegal use and application, it is necessary to be with the supervision of the state. 4. In general, the cultivation, production, sale of cannabis and its similar, which have a legal and illegal use, has no issue for the purpose of legal use. However, if there is a chance of abuse, its use must take place with the supervision of the state and if there is a law in relation to this, it must be respected. 5. Hashish is not impure. 6. No. 7. It does not harm its prayer. 8. No.
Mohammad ‘Alavi Gorgani	1. Cannabis consumption is accepted but its derivatives, if harmful or confusing the mind, are haram. 2. Has no issue, as long as necessary. 3. Has no issue. 4. Has no issue. 5. It’s not impure. 6. If it’s smoked, it breaks the fasting of Ramadan. 7. It’s valid. 8. It’s against the law and in many cases it’s haram.
Hossein Mazaheri Esfehani	1, 2, 3, 4. The use of hashish and its production is not permitted.
	5. Cannabis is not impure.
	6. If a Muslim uses hashish during Ramadan, that breaks fasting.
	7. If the Muslim is conscious while on cannabis the prayer is valid. But if he is intoxicated the prayer is invalid.
	8. It’s not valid.
Mohammad-Reza Modaressi Yazdi	1. Cannabis itself is allowed. Its derivatives are not. If you obtain a composition of hashish, then it is not allowed, as in the case of grapes and wine. 2. It is *haram* and sinful. 3. Its cultivation for the production of hashish is not allowed under any condition. 4. It’s not allowed. 5. It is not impure. 6. It is with no doubt haram and it’s necessary to give up for Ramadan. 7. It is a big sin like drinking wine or incest, but his prayer is valid if he/she is not intoxicated. 8. Advertising prohibited products is not allowed and is sinful.
Ali Sistani	1. Cannabis use in itself in not haram. Its derivatives are different, if they are intoxicant/inebriant (*musakkar*) or are harmful for sure, then they are haram. 2. If it’s among the forbidden one then it’s not permitted, unless in the time of necessity and urgency and in the measure necessary to solve the urgency. 3. Yes, it is allowed. 4. Yes, it’s allowed. 5. It’s not impure. 6. For the sick for whom not using it is harmful, then it is not forbidden to use it. 7. It is valid but if its derivate is haram, he/she should repent. 8. Advertising its legal use is not forbidden.

## Footnotes

1 One has to consider that the Shi‘a clergy operates transnationally and can express legal opinions that apply to Shi‘a believers wherever they are. In the case of Iraq-based marja‘s they often happen to be involved culturally and politically in Iranian affairs. This is the case of prominent cleric Ali al-Sistani, himself an Iranian residing in Iraq, see (Louër 2008). Transnational Shia politics: religious and political networks in the Gulf. Columbia University Press.

2 There is no appointment process for *marja‘s*. They reach legitimacy based on the number of their followers and on their record of publication. See (Clarke 2007).

3 It applies to other contexts too: the influence of Pentecostalism in Latin American drugs policy; Evangelical churches’ ideas about substance disorder in the United States; Orthodox Christianity and its leverage over Russian policymaking. See Zigon, J. (2011). "A moral and ethical assemblage in Russian Orthodox drug rehabilitation." Ethos**39**(1): 30–50, Seward Delaporte, P. (2017). "‘We Will Revive’: addiction, spiritual warfare, and recovery in Latin America’s cocaine production zone." Third World Quarterly: 1–16.

4 There are ways to get around apparent incongruences when the necessity of public law outplays religious dogmas In special cases − and drugs policy is one of these – the laws are not discussed neither by the Parliament nor by the Guardian Council. They are discussed, formulated and legislated in the Expediency Council. This body has the power to legislate in situations when Parliament and Guardian Council do not find a way out of the legislative impasse. It is also charged with the duty to formulate laws on issues of highest interest to the political order. Since 1988, the date of the first drugs laws in Iran, the Expediency Council has been the sole institution charged with the task of promulgating drug laws Ghiabi, 2018.

5 It is problematic to compare the response of the *marja‘s* with that of scholars in other jurisdiction, since the role of the *marja‘s* is of exegesis rather than systematic jurisprudence – at least in the circumstances of this study. To do so, one would require a comparative approach, which in fact could be the object of a future study.

6 The debate about cannabis legalisation is paralleled and in fact contextual to the debate about legalisation of opium. Iran already disposes of a controlled programme of opium tincture, but public officials have voiced support for the introduction of a medical opium programme, similar to the one adopted during the 1970s under the Pahlavi monarchy. For today’s debate, see ADNA 12 September 2017. For an overview of the 1970s programme, see Ghiabi, 2018a.
